# Data quality and auditing within the Netherlands Heart Registration: using the PCI registry as an example

**DOI:** 10.1007/s12471-022-01752-1

**Published:** 2023-01-16

**Authors:** S. Houterman, A. van Dullemen, M. Versteegh, W. Aengevaeren, P. Danse, E. Brinkman, D. Schuurman, D. van Veghel, PCI Registration Committee of the Netherlands Heart Registration

**Affiliations:** 1 Netherlands Heart Registration, Utrecht, The Netherlands; 2 Department of Cardiology, Onze Lieve Vrouwe Gasthuis Hospital, Amsterdam, The Netherlands; 3 Department of Cardiology, Rijnstate Hospital, Arnhem, The Netherlands; 4 Department of Medical Informatics, Amsterdam UMC, Amsterdam, The Netherlands

**Keywords:** Data quality, Audit, Percutaneous coronary intervention, Netherlands Heart Registration

## Abstract

**Aim:**

The aim of this article is to present the method and results of the data quality control system and au﻿dit within the Netherlands Heart Registration (NHR) using data of patients treated with percutaneous coronary intervention (PCI) in the Netherlands as an example.

**Methods:**

The NHR is a Dutch nationwide registry of all cardiac interventions, comprising data from all 71 hospitals, of which 30 are cardiac intervention or heart centres. Each year, within the NHR, data validation and verification is performed by standard quality controls and monitoring visits (audits). For the audit in 2019, a sample of 50–100 medical records of patients treated with PCI in 2016 and 2017 were reviewed in each hospital by an independent audi﻿tor. The data received by the NHR were compared with the information in the hospitals’ medical records. In to﻿tal 12 patient characteristics, 5 intervention variables and 3 outcome variables were screened. The value of a variable was considered discrepant if more than 10% of the medical records reviewed regarding this variable were not consistent with the reported data received by the NHR.

**Results:**

For all variables together, the consistency was high, 97.6%. All variables, except multivessel disease (9.3% discrepancy in the 2622 medical records reviewed), had an accuracy above 95%.

**Conclusion:**

The results of the audit of the PCI medical records show that the overall quality of the data is high. For variables such as multivessel disease it is important to improve knowledge of the definitions and to train all those involved in the registration process.

**Supplementary Information:**

The online version of this article (10.1007/s12471-022-01752-1) contains supplementary material, which is available to authorized users.

## What’s new?

- The Netherlands Heart Registration (NHR) has a solid data quality assurance system with different kinds of data quality checks.
- Each year data validation and verification is performed by means of audits.
- Overall, the quality of data in the PCI registry of the NHR is high.
- For real-world data of the NHR to be used for improvements in quality of care and scientific research, it is important that these data are reliable and accurate.

## Introduction

Cardiovascular diseases are the leading cause of death in the Western world, accounting for an estimated 17.9 million lives each year [1]. Increasing healthcare costs and an aging population force society to make the ‘right’ decisions. Therefore, it is important to have insight into the treatment outcomes of patients with cardiovascular disease over time. Measuring treatment outcomes over time enables continuous improvement of treatment results and sharing of best practices among hospitals [2]. Public reporting of real-world data from quality registries producing benchmark data showed that this contributes to improvement in the quality of care [3, 4]. A well-maintained nationwide registry can provide real-world data as a source for clinical research. This is in addition to controlled clinical trials that adhere to various exclusion criteria and in general are at risk of selecting populations that do not represent daily practice [5]. Worldwide there is a trend towards registration and monitoring of healthcare outcomes. The value-based healthcare (VBHC) theory has raised the ambition to measure and improve outcomes that matter most to patients, also within the cardiovascular field [6]. Renowned initiatives are the International Consortium of Health Outcomes Measurement (ICHOM) [7], the Netherlands Heart Registration (NHR) and SWEDEHEART [8, 9].

When using real-world data from a large nationwide registry, it is of utmost importance that these data are reliable and accurate. Within the NHR a quality system is used in which quality control and auditing are important parts [10]. The aim of this article is to present the method and results of the data quality control system and audit within the NHR using data of patients suffering from coronary artery disease treated with percutaneous coronary intervention (PCI) in the Netherlands as an example.

## Methods

### Netherlands Heart Registration

The NHR is a Dutch nationwide, physician-driven and patient-focused quality registry. The main objective is to facilitate and improve quality of care by registering, analysing and providing relevant information on cardiovascular care. To monitor and improve the quality of cardiovascular care, all 71 Dutch hospitals upload data to the NHR (30 of which are cardiac intervention centres, 16 thereof with cardiac surgery). For each intervention or operation, information on patient characteristics, procedural and outcome data are registered.

Data collection and registration are performed by the participating hospitals in their electronic medical records, extracted and submitted to the NHR in a secure online environment and transferred in an encrypted format to a central server.

The Dutch Society of Cardiology and the Dutch Society of Cardiothoracic Surgery are both strongly connected with the NHR. As a part of their quality policy or as a legal obligation, both societies decide which sets of variables and publications on outcomes are mandatory for their members. An example of a legal obligation is the standard set of variables for PCI that is part of the transparency calendar, a mandatory register in the Netherlands with the aim of creating insights into quality of care for a selection of diseases [11]. Within the NHR, hospitals can participate in a voluntary VBHC programme in which patient-relevant outcomes corrected for patient characteristics are presented yearly in a publicly accessible report [12, 13].

### Data quality assurance

**Fig. 1:**
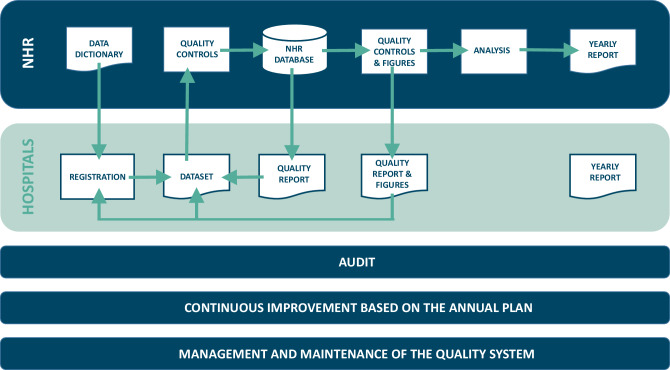
Flowchart of the Netherlands Heart Registration (*NHR*) NEN-7510 certified data quality assurance system

Within the NHR a Royal Netherlands Standardization Institute (NEN) 7510 certified data quality assurance system is used (Fig. 1). To ensure the validity and consistency of the data, the NHR provides a detailed data manual [14], which contains the definitions of all the variables collected in the NHR and contains coding guidelines for data collection.

Data in the NHR are validated using different processes, giving the hospitals the opportunity to verify the uploaded data before being used for public reporting or scientific research. After uploading of the data, several automated quality controls are carried out. Through defining several requirements for uploading, a first ‘wall’ is created for error checking (for example, if identifying variables such as the intervention date are missing). The hospitals receive an automated quality report from the system containing information about failures or inconsistencies in the data. There are several data quality checks: (1) missing values, (2) domain errors and (3) logical controls based on medical expertise. In addition, hospitals receive a factsheet with a summary of the most important characteristics and the completeness of the uploaded data. Thereafter, the data analysts of the NHR conduct supplemental quality checks and maintain a spreadsheet (Excel, Microsoft, Redmond, WA, USA) of the uploaded data with percentages and corresponding confidence limits for each variable, as well as the percentage of missing data, for each hospital separately. These overviews give the hospitals the opportunity to check the data for completeness, outliers and if necessary make data corrections. Within the NHR only data that are more than 90% complete are publicly presented. Finally, the draft figures for the yearly report are presented to the hospitals for a final check.

Audits constitute a part of the data quality assurance system within the NHR. Each year data validation and verification is performed by monitoring visits (audits). During the audit, a selection of the data that was submitted to the NHR is compared with the information in the hospitals’ medical records. This selection of variables is based on clinical importance and variables that are probably susceptible to differences in interpretation of the definitions by different hospitals. An independent nurse practitioner, employed by the NHR, visits the hospitals each year and a selection of data submitted to the NHR is compared with the information in the hospitals’ medical records. Two dedicated medical specialists (a cardiothoracic surgeon and a cardiologist) are members of the audit team. The medical specialists visit a random sample of hospitals each year together with the nurse practitioner to supervise the audit and, in addition, they can be consulted for difficult cases or discussions within the hospitals.

For the audit in 2019, a selected sample of 50–100 medical records of patients treated with PCI in 2016 and 2017 were reviewed in each hospital. The random sample comprised 1/3 medical records of deceased patients, 1/3 medical records of high-risk patients and 1/3 medical records randomly selected from the whole database. In total, 12 patient characteristics [age, serum creatinine, diabetes mellitus, multivessel disease, chronic total occlusion of the coronary artery, history of myocardial infarction, indication for PCI, cardiogenic shock, out-of-hospital cardiac arrest and previous coronary artery bypass grafting (CABG) or PCI], 5 intervention variables (heart team meeting, date of intervention, access route, treated vessel and type of intervention) and 3 outcome variables [urgent CABG within 1 day, myocardial infarction within 30 days, target vessel revascularisation within 1 year] were screened. We selected from the variables mandatory for the transparency calendar and the VBHC programme.

The value of a variable was considered discrepant if more than 10% of the medical records reviewed on this variable were not consistent with the reported data received by the NHR. Variables with more than 10% discrepancy will be reviewed in a random sample of patients in the year after each audit.

### Data analysis

Descriptive statistics were performed and data are presented as numbers and percentages. No formal statistical analyses were performed.

## Results

### Data quality

More than 40,000 PCI procedures are performed in the Netherlands each year [10]. The total number of coronary arteries treated in 2019 was 52,498. In 2019, the published data of the NHR were 99.4% complete (range 91.7–100%) for patient characteristics, 99.5% (range 87.9–100%) for intervention variables and 99.4% (range 86.7–100%) for outcome variables (Tab. 1).

**Table 1:** Overview of the completeness of the mandatory variables within the PCI registry of the Netherlands Heart Registration (year 2019)

	Number	Percentage
*Identifying variables*		
Age	40,712	100.0%
Gender	40,712	100.0%
*Patient characteristics*		
Creatinine	39,640	97.4%
Diabetes	40,418	99.3%
Dialysis	40,642	99.8%
Multivessel disease	40,598	99.7%
Chronic total occlusion	40,673	99.9%
Previous MI	40,447	99.3%
Indication PCI	40,666	99.9%
Shock	40,676	99.9%
OHCA	40,689	99.9%
Previous PCI	40,582	99.7%
Previous CABG	40,565	99.6%
*Intervention variables*		
Date of intervention	40,712	100.0%
Heart team	40,695	100.0%
Access route	40,636	99.8%
PCI vessel	40,664	99.9%
PCI type	40,661	99.9%
*Outcome variables*		
Acute CABG (≤ 24 h)	40,699	100.0%
MI (≤ 30 days)	33,935	83.4%
Target vessel revascularisation (≤ 1 year)	35,638	87.5%
Staged procedure	30,324	74.5%
Mortality	40,527	99.5%
Date of death	40,526	99.5%

*MI* myocardial infarction, *PCI* percutaneous coronary intervention, *OHCA* out-of-hospital cardiac arrest, *CABG* coronary artery bypass graft

Some examples of data quality checks that are performed within the NHR database are given in Tab. 2. An example of a factsheet with a summary of the most important characteristics and the completeness of the uploaded data is given in Fig. S1 (Electronic Supplementary Material). An example of the Excel overview of the uploaded data with percentages and corresponding confidence limits for each variable, as well as the percentage of missing data, for each hospital separately, is given in Fig. S2 (Electronic Supplementary Material). Separate dispersion charts with percentages for each variable according to hospital are presented in Fig. S3 (Electronic Supplementary Material). In these figures the overall percentage across the hospitals with the corresponding 95% confidence intervals (lower and upper limit) are presented.

**Table 2:** Overview of data quality checks in the PCI registry of the Netherlands Heart Registration after uploading of the dataset

Type of control	Examples
*Missing values*	Variables that are coded as missing values, due to missing data
	Correct missing values: missing date of complication because there was no complication
	Missing values for left ventricular ejection fraction are only allowed if PCI indication is STEMI or non-STEMI
*Domain error*	Age: should be between 0 and 120 years
	Intervention number for the conducted intervention should be unique across years
	Implantation codes not uploaded according to specifications
*Logical controls*	A patient with out-of-hospital cardiac arrest cannot have the indication elective
	It is unlikely that a patient with a chronic total occlusion is a STEMI patient
	If a patient died, the date of target vessel revascularisation should be before the date of death

*PCI* percutaneous coronary intervention, *STEMI* ST-elevation myocardial infarction

### Audit

**Fig. 2:**
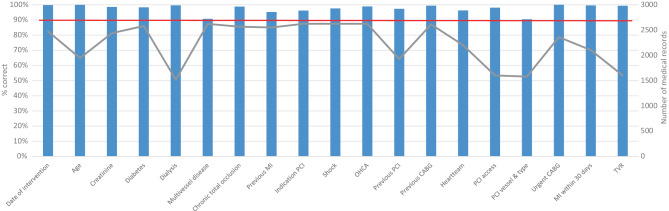
Overview of the consistency per variable (in % on the left *y*-axis in the *blue bars*) and the total number of medical records of PCI patients reviewed in 2019 (in numbers on the right *y*-axis and the *grey line*) within all hospitals (*red line* is the 90% acceptance border). *MI* myocardial infarction, *PCI* percutaneous coronary intervention, *OHCA* out-of-hospital cardiac arrest, *CABG* coronary artery bypass graft, *TVR* target vessel revascularisation

In 2019, a total of 16 heart centres and 13 PCI centres were visited. The results of the audit within the PCI registry for all variables together showed that consistency was high, 97.6% (Fig. 2). All variables, except multivessel disease (9.3% discrepancy of the 2622 medical records reviewed) and PCI vessel and type (9.5% discrepancy of the 1579 medical records reviewed), had an accuracy above 95%.

In Tab. 3 the number and percentage of hospitals with more than 90% consistency per variable are shown. In total, 12 out of 29 hospitals (41%) showed more than 90% consistency for the variables audited. In almost 30% of the hospitals there was > 10% discrepancy in the variables multivessel disease and PCI type.

**Table 3:** Number of hospitals (total *N* = 30) with more than 90% consistency per variable for the patients undergoing percutaneous coronary intervention (*PCI*)

Variable	Number (%) of hospitals > 90% correct
Multivessel disease	20 (69%)
PCI type	21 (72%)
Previous myocardial infarction	24 (83%)
Heart team	26 (90%)
Previous PCI	26 (90%)
PCI vessel	27 (93%)
Indication PCI	28 (97%)

## Discussion

The results of the audit of the PCI medical records and the quality checks of the NHR show that the overall quality of data is high within the PCI registry. For variables such as multivessel disease it is important to improve knowledge of the definitions and to train all those involved in the registration process.

The hospitals are responsible for the quality of the data uploaded to the NHR. Through presenting quarterly quality checks of the PCI data to the registration committee, doctors can discuss the quality of the data. In this discussion the results of the possible discrepancies in the outcome data are most prominent. Moreover, the doctors in the PCI registration committee discussed the results of the PCI audit. In particular, the definition of multivessel disease was reviewed. The auditor of the NHR presented several difficult cases to the registration committee, and the way in which the variables should be recorded was discussed with the members. Based on this discussion the definition of multivessel disease was modified and the presented cases were added to the data dictionary of the NHR. More attention was paid in the hospitals to registration of multivessel disease according to the modified definition (see Electronic Supplementary Material). Based on more recent data the distribution of this variable was more equal across the hospitals, showing an improvement in the consistency of this variable. For indication of PCI two extra categories were added (elective for angina pectoris and elective for staged procedure). For cardiogenic shock an additional pilot registry was started. Based on the results of this project the registration committee will analyse if it is necessary to reconsider the definition of this variable and/or to add some additional relevant variables to the PCI registry.

In SWEDEHEART monitoring visits are carried out yearly in approximately 20 randomly selected hospitals [9]. Data entered into the database of SWEDEHEART are compared with the information in the medical records of 30–40 randomly chosen patients for each hospital. In 2007, 637 computer forms from 21 hospitals containing 38,121 variables were reviewed and 96.1% (range 92.6–97.4%) agreement was found.

The National Cardiovascular Data Registry in the United States has a data quality programme which consists of three main components: data completeness, consistency and accuracy [15]. Within each registry, 300–625 records are audited annually at 25 randomly identified sites. In 2010, the raw accuracy of data in the CathPCI Registry was 93.1% (range 89.4–97.4%).

In the Chinese Cardiac Surgery Registry, comprising 46,303 surgical procedures at 87 participating centres, a routine data quality audit was performed in the period 2013–2015 [16]. In a randomly selected sample of 5–10% of the medical records from each site, the completeness and accuracy rates (using a rate of data accuracy greater than 95%) were 97.6% and 95.1% respectively.

Within the NHR we continuously evaluate and improve our processes using a PDCA cycle. Constant monitoring and improving of quality of care is of utmost importance for patients and healthcare providers. The PCI registration committee started several initiatives in order to create new relevant insights. For example, an additional pilot registry was initiated for patients with cardiogenic shock at the start of PCI and a registry for isolated and PCI-combined diagnostic intracoronary procedures. Besides, additional analyses have been proposed in order to create more in-depth insight into subgroups of patients. One example is the analyses of the variation in the revascularisation strategy in patients with multivessel disease, as the optimal revascularisation strategy is debatable (multivessel PCI vs culprit-only PCI), especially for patients with non-ST-elevation myocardial infarction. Analyses of inter-physician variability in addition to the common benchmark analyses on a centre level are also of increasing interest.

Modern healthcare, with the digitisation of clinical information, is reshaping clinical research [17]. Re-using real-world data from routine clinical practice has become an integral component of research within the cardiovascular field and could accelerate implementation of these results in practice. Several registry-based randomised clinical trials have been published using the data from SWEDEHEART [18, 19], showing the added value for healthcare of these types of trial using already existing real-world data. In future years, the HEART4DATA consortium (in which the NHR is participating) will create a national and sustainable infrastructure for cardiovascular registry-based research in the Netherlands. This infrastructure will develop a framework for the governance, ethical, legal, financial, IT and methodological factors needed for registry-based research.

In the next few years we will further audit the different NHR registries by comparing the uploaded data with the original patient data registered in their medical records.

## Supplementary Information

PCI Registration Committee members of the Netherlands Heart Registration

Fig. S1 …

Fig. S2 …

Fig. S3 …

Definition multivessel disease
