# Supplementary material for: Data quality and auditing within the Netherlands Heart Registration: using the PCI registry as an example
Source: Neth Heart J. 2023 Jan 16;31(9):334–9. doi: 10.1007/s12471-022-01752-1 (PMC10444924; doi:10.1007/s12471-022-01752-1)
Supplement: Supplementary file 1 — PCI Registration Committee members of the Netherlands Heart Registration [file 12471_2022_1752_MOESM1_ESM.docx]

**Addendum**

The following physicians are the members of PCI Registration Committee of the NHR. They represent the hospitals that have provided data for the PCI registry.

| \| M \| Scholte \| Albert Schweitzer Hospital \| \| --- \| --- \| --- \| \| M \| Meuwissen \| Amphia \| \| JP \| Henriques \| Amsterdam University Medical Centre, University of Amsterdam \| \| KMJ \| Marques \| Amsterdam University Medical Centre, VU Medical Centre \| \| T \| Teeuwen \| Catharina Hospital \| \| H \| Al Hashimi \| Canisius Wilhelmina Hospital \| \| M \| Magro \| Elisabeth-TweeSteden Hospital \| \| J \| Daemen \| Erasmus Medical Centre \| \| BJ \| Sorgdrager \| Haaglanden Medical Centre \| \| CE \| Schotborgh \| Haga Hospital \| \| V \| Roolvink \| Isala \| \| J \| Polad \| Jeroen Bosch Hospital \| \| I \| Karalis \| Leiden University Medical Centre \| \| M \| van der Ent \| Maasstad Hospital \| \| AJW \| van ‘t Hof \| Maastricht University Medical Centre and Zuyderland Medical Centre \| \| F \| Spano \| Meander Medical Centre \| \| J \| Brouwer \| Medical Centre Leeuwarden \| \| MG \| Stoel \| Medical Spectrum Twente \| \| A \| Dedic \| Noordwest Hospital Group \| \| G \| Amoroso \| OLVG \| \| C \| Camaro \| Radboud University Medical Centre \| \| PW \| Danse \| Rijnstate \| \| JP \| van Kuijk \| St. Antonius Hospital \| \| EK \| Arkenbout \| Tergooi \| \| WT \| Ruifrok \| Treant Zorggroep, Scheper Hospital \| \| A \| Kraaijeveld \| University Medical Centre Utrecht \| \| E \| Lipsic \| University Medical Centre Groningen \| \| S \| Aydin \| VieCuri Medical Centre \| \| R \| Erdem \| Zorgsaam Hospital \| |
| --- | --- | --- | --- | --- | --- | --- | --- | --- | --- | --- | --- | --- | --- | --- | --- | --- | --- | --- | --- | --- | --- | --- | --- | --- | --- | --- | --- | --- | --- | --- | --- | --- | --- | --- | --- | --- | --- | --- | --- | --- | --- | --- | --- | --- | --- | --- | --- | --- | --- | --- | --- | --- | --- | --- | --- | --- | --- | --- | --- | --- | --- | --- | --- | --- | --- | --- | --- | --- | --- | --- | --- | --- | --- | --- | --- | --- | --- | --- | --- | --- | --- | --- | --- | --- | --- | --- | --- |
