# Supplementary material for: Data quality and auditing within the Netherlands Heart Registration: using the PCI registry as an example
Source: Neth Heart J. 2023 Jan 16;31(9):334–9. doi: 10.1007/s12471-022-01752-1 (PMC10444924; doi:10.1007/s12471-022-01752-1)
Supplement: Supplementary file 5 — Definition multivessel disease [file 12471_2022_1752_MOESM5_ESM.docx]

**Supplementary file**

Old definition multivessel disease

Presence of multivessel disease at the time of current intervention.

At first intervention: if at least 70% stenosis in 2 or more native coronary arteries (right coronary arterie (RCA) / ramus descendens anterior (RDA) / ramus circumflex artery (RCX), or in the first order side branches).

In patients who have already undergone a PCI and/or CABG: if at least 70% stenosis in 1 or more native coronary arteries that have not been previously revascularized (RCA / RDA / RCX, or in the first order side branches) and/or there was multivessel disease during a previous intervention.

Note: A main stem stenosis counts as multivessel disease.

New definition multivessel disease

Presence of multivessel disease at the time of current intervention.

At first intervention: if at least 70% stenosis in 2 or more native coronary arteries (RCA / Left Anterior Decending (LAD) / RCX, or in the first order side branch with a diameter of at least 1.5 mm).

In patients who have already undergone a PCI and/or CABG: if at least 70% stenosis in 1 or more native coronary arteries that have not been previously revascularized (RCA / RDA / RCX, or in the first order side branch with a diameter of at least 1.5 mm) and/or there was multivessel disease during a previous intervention.

NOTE-1: If functional measurements are done (FFR, iFR) these determine the significance of the stenosis and not the angiographic estimation of the stenosis (> 70%); the stenosis is found to be significant at an FFR ≤ 0.80 and an iFR ≤ 0.89.

NOTE-2: A significant main stem stenosis (angiographic estimate of the stenosis > 50% and/or an FFR ≤ 0.80 and an iFR ≤ 0.89) counts as multivessel disease.
